# *In situ* dissolution of the international simple glass (ISG-1 & ISG-2) and UK high-level-waste glass in extreme γ-radiation environments

**DOI:** 10.1557/s43580-025-01268-x

**Published:** 2025-04-30

**Authors:** Adam J. Fisher, Clare L. Thorpe, Latham T. Haigh, Sarah E. Pepper, Ruth Edge

**Affiliations:** 1 Dalton Cumbrian Facility, The University of Manchester, Westlakes Science Park, Moor Row, Cumbria, CA24 3HA UK; 2 Department of Materials Science & Engineering, The University of Sheffield, Sheffield, South Yorkshire S1 3JD UK

## Abstract

*In situ* γ-irradiated dissolution of the International Simple Glass (ISG) 1 & 2 and a UK high-level waste glass (post-operational-clean-out (POCO)) was investigated following a modified Product Consistency Test-B protocol for 158 d at 40 °C in ultra-high-quality water. Tests were conducted under atmospheric conditions and received a total dose of 21.6 MGy delivered at a rate of 0.137 MGy d^–1^. The normalised mass loss of B, Na, Ca and Mg were slightly higher in γ-irradiated tests when compared to non-irradiated tests whilst the normalised mass loss of Si was comparable or slightly lower. Boron-normalised mass losses of 0.87 ± 0.31, 0.60 ± 0.04 and 0.68 ± 0.07 g m^–2^ were calculated for γ-irradiated ISG-1, ISG-2 and POCO, respectively, whilst normalised mass losses in non-irradiated controls were 0.62 ± 0.01, 0.57 ± 0.01 and 0.41 ± 0.06 g m^–2^. The difference was tentatively attributed to acidification during irradiation.

**Graphical abstract:**

**Figure:**
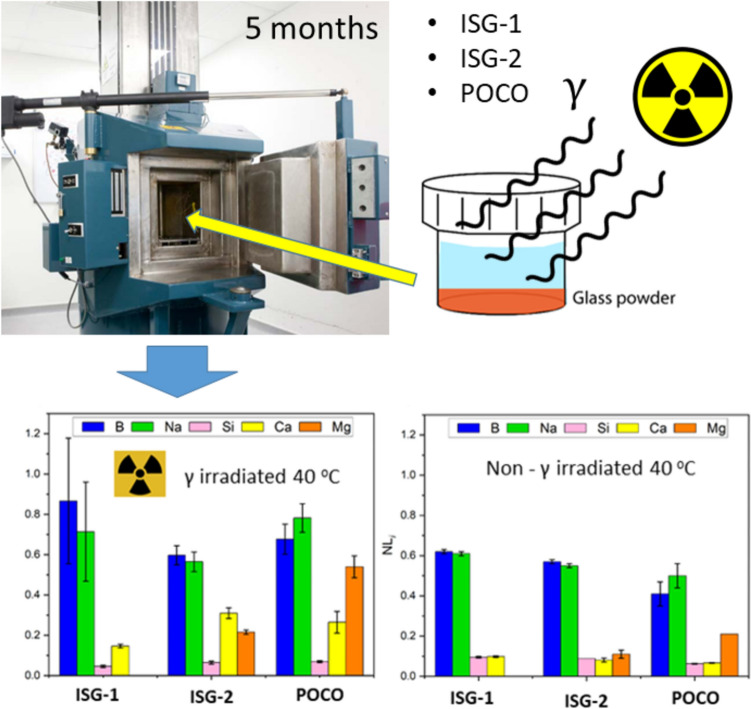
*In situ* γ-irradiation tests on HLW reference glasses

## Introduction

As radioactive waste disposal programmes continue to develop the safety case for deep geological repositories, renewed interest into the effects of radiation on the structure and chemical durability of vitrified high-level-waste (HLW) has gathered momentum [1–7].

Radiation (α, β and γ) can detrimentally affect borosilicate HLW glass dissolution in two major ways: 1) it can lead to structural or chemical changes, including oxygen migration [8], and 2) it can lead to changes in the leachate chemistry due to radiolysis [1, 9–11].

It is hypothesised that absorption of γ-radiation by electrons in the glass structure can lead to the breaking of chemical bonds, the appearance of unbound ions and the formation of free oxygen in the glass, which can impact its properties and aqueous durability [12]. Studies on the effect of γ-irradiation alone have reported mixed results. Some recent studies report negligible effects, such that the dissolution behaviour of pristine complex and simple HLW glasses in non-irradiated tests and the same glasses γ-irradiated with doses ranging from 2 to 200 MGy are near identical [4, 13]. However, it should be noted that the self-γ-irradiation dose to HLW glass after 10,000 years is expected to be ~ 2,000 MGy [12]. Other studies report small but measurable differences (e.g. [14]) where increased leaching was attributed to acidification during radiolysis. High doses of γ-radiation may cause the formation of radiation-induced nanoparticles, such as sodium metal colloids [15], which may influence the chemical durability. As such, future work is expected to explore these challenges and address remaining questions relating to the response of HLW glass to radiation. In particular, integrated studies evaluating the coupling effect between radiation and the environmental materials expected to interact with HLW glass in the repository will be further developed. Prior to these future studies, dissolution data on internationally recognised reference glasses, such as the International Simple Glasses (ISG-1 & ISG-2) will always serve as a useful baseline. This study on ISG-1, ISG-2 [16] and an inactive UK post-operational-clean-out (POCO) HLW glass with a high MoO_3_ content [17–19] provides benchmark *in situ* γ-irradiation dissolution data (158 d, ~ 40 °C, external γ-irradiation source up to 21.6 MGy), which can also be used as input for, and to validate anticipated numerical dissolution models for these glasses. Results from control dissolution tests (at 20 °C and 40 °C) conducted on the same glasses but without external γ-irradiation are also presented.

### Experimental details

*In situ* γ-irradiation dissolution tests were conducted following a modified Product Consistency Test-B (PCT-B) methodology ASTM C1285-21 [20]. Powders (75–100 µm diameter) were prepared from three glasses: ISG-1 [16] (0.590 g; density 2.51 g cm^–3^), ISG-2 [16] (0.578 g; density 2.46 g cm^–3^) and POCO [18] (0.712 g; density 3.05 g cm^–3^) and leached in sealed polypropylene vessels using 8 mL of ultra high-quality (UHQ) water (18.2 MΩ cm at 25.0 °C, pH_RT_ 7.01) to attain the desired geometric glass surface area to solution volume ratio (SA/V) of 2,000 m^–1^. A small wafer of each sample (~ 3 × 2 × 1 mm, 1 µm polished surface finish) was inserted on top of the glass powders to enable post-dissolution analysis of alteration layers. Such insertions had negligible effect on SA/V.

**Fig. 1:**
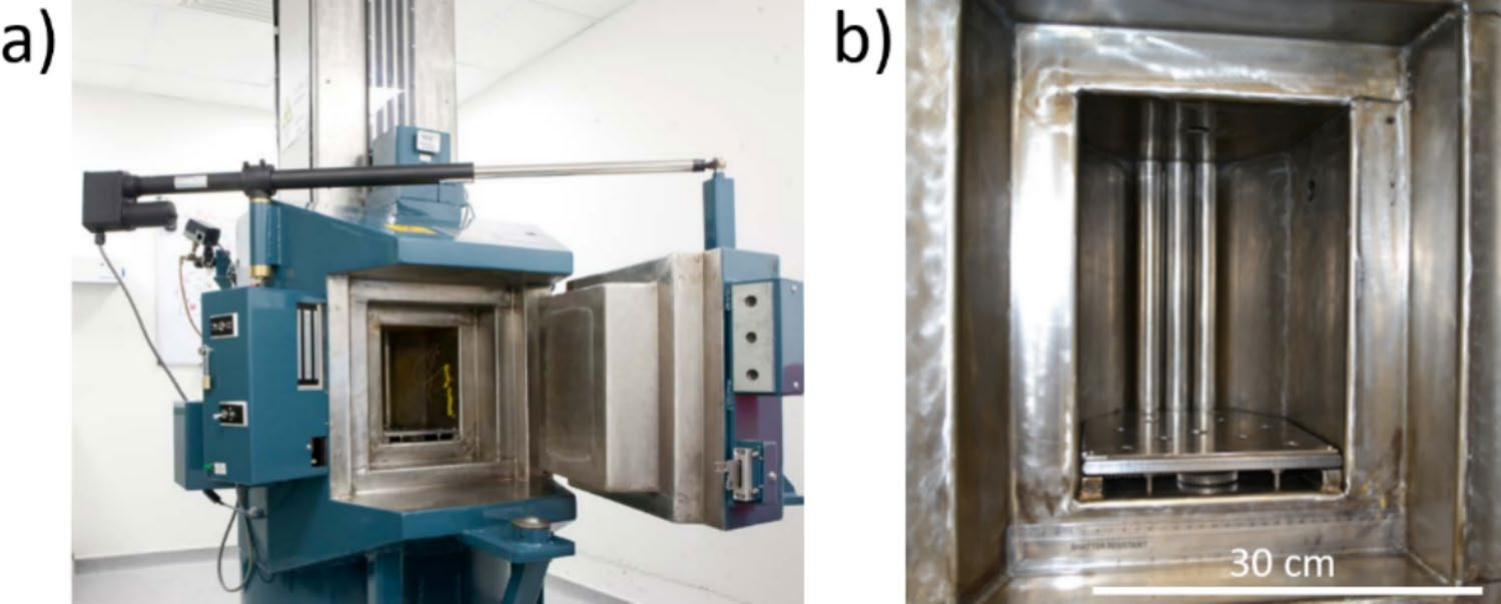
The foss therapy services 812 Co-60 γ-irradiator at the dalton cumbrian facility (UK): **a** overview; **b** close-up of the chamber [21]

The external γ-radiation was provided from a Co-60 (1.17–1.33 MeV) source (Foss Therapy Services 812) located at The Dalton Cumbrian Facility (UK) [21] (Fig. 1). Absorption of γ-radiation by the Pb walls of the irradiator means that the temperature within the chamber reaches approximately 314 K (41 °C) after the first 45 min of each irradiation, whereupon it remains stable throughout the γ-irradiation [15]. The *in situ* PCT-B dissolution tests were duplicated and conducted under atmospheric conditions for 158 d (3,792 h) and were γ-irradiated at a rate of approximately 0.134 MGy d^–1^ (5.6 kGy hr^–1^). Irradiation was not continuous but staged over 78 irradiator sessions. There were short periods of time, (0.25–12 h) between irradiator sessions where dissolution proceeded without external γ-irradiation, during which the temperature likely reduced to ~ 22.0 °C (room temperature). A realistic dose rate of 0.05 kGy hr^–1^ is expected at the time of groundwater contact in the French geological disposal concept [4, 22]. Total doses of 21.57, 21.27 and 21.11 MGy were received for the ISG-1, ISG-2 and POCO dissolution tests, respectively. Duplicate blank UHQ tests were also conducted. Counterpart non-irradiated PCT-B tests were conducted in duplicate on all glass samples and blanks to enable a baseline comparison in UHQ at 40 °C under atmospheric conditions. Both controls and irradiated experiments used identical glass from the same batch, the same SA/V ratio, and used the same ICP-OES instrument and operator to analyse aqueous solution data. However, glasses were size reduced at different laboratories and incubated in different vessels (controls were prepared at the University of Sheffield in 15 ml PTE vessels whereas irradiated experiments were prepared at Dalton Cumbrian facility and incubated in polypropylene vessels).

Dissolution tests were subjected to one sampling time point (158 d) where a 4 mL aliquot of each sample was taken for inductively coupled plasma optical emission spectrometry (ICP-OES) and mass spectroscopy (ICP-MS) analysis to determine the concentration of elements (C_*i*_) in solution. An additional 1 mL aliquot was taken to determine the pH_RT_. Dissolution was measured from the normalised mass loss of glass based on the release of element *i* (g m^–2^) (NL_*i*_) according to: NL_*i*_ = (C_*i*_–C_*i*,b_) / (f_*i*_ [SA/V]).

where C_*i*_ and C_*i,b*_ are the average concentration of element *i* in the leachate and blank tests, respectively (mg L^−1^), measured using ICP-OES (Thermofisher, iCAP Duo) and ICP-MS (Thermofisher, iCAP RQ ICP); f_*i*_ is the mass fraction of *i* (unitless) and SA/V is the surface area of the total particulates to volume of solution (m^−1^), based on the geometric surface area. Uncertainty in NL_*i*_ was calculated by the standard deviation of the sum of uncorrelated random errors associated with C_*i*_, C_*i,b*_, f_*i*_ and SA/V.

Post-dissolution, glass wafers and powders were epoxy mounted, prepared in cross section, polished to a 1 µm surface polish, and gold coated for scanning electron microscopy (SEM) analysis using an FEI Quanta 250 microscope. Whole glass powders post-dissolution were adhered to carbon tabs, gold coated, and were also analysed by SEM.

## Results and discussion

**Fig. 2:**
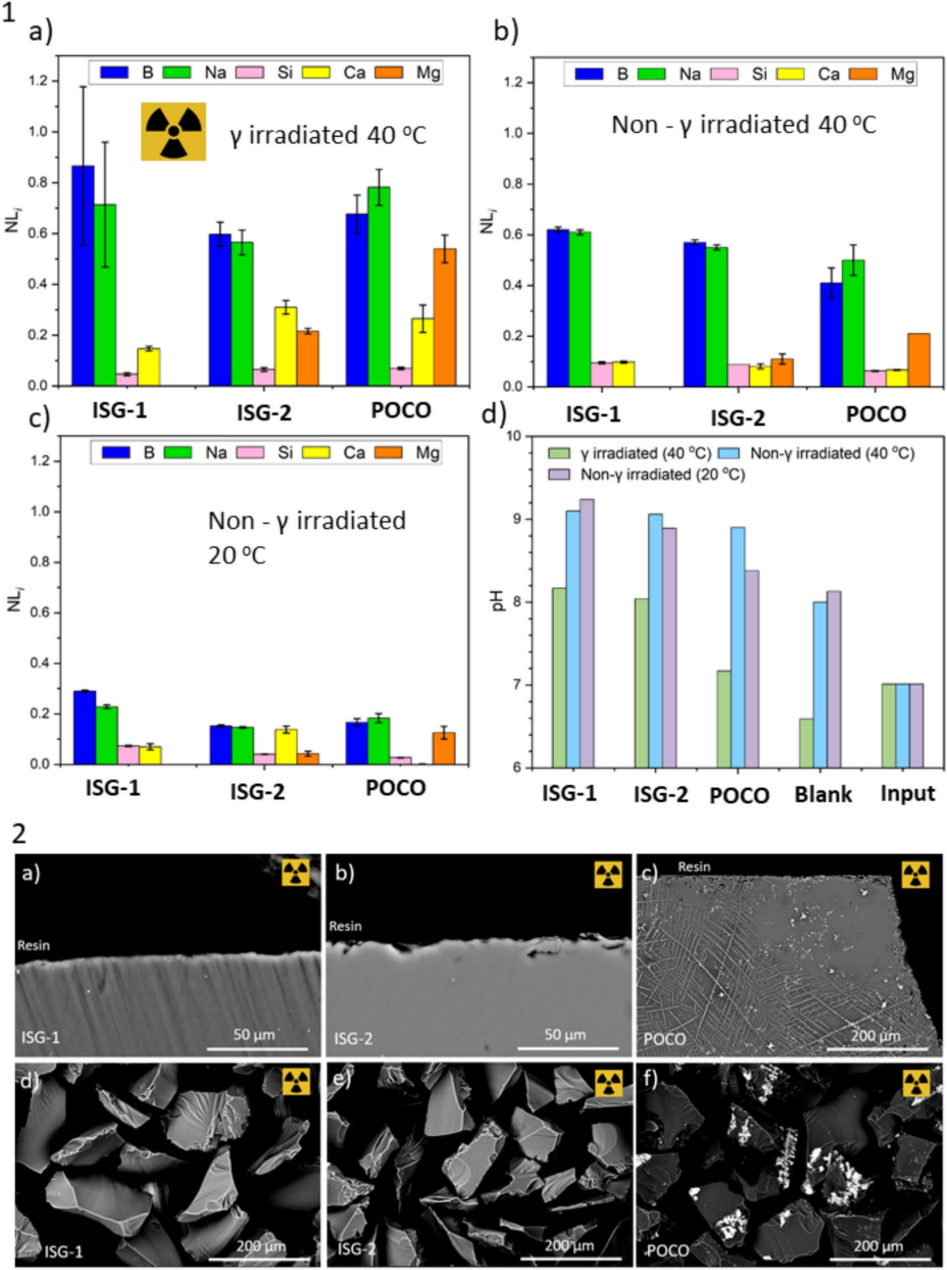
Part 1: Bar graphs of **a** NL_i_ from *in situ* γ-irradiation dissolution tests after 158 d at ~ 40 °C; **b** NL_i_ from baseline non-irradiation dissolution tests at 40 °C after 158 d; **c** NL_i_ from baseline non-irradiation dissolution tests at 20 °C after 158 d; and **d** comparison of the pH_RT_ (± 0.1) from all dissolution tests after 158 d. Part 2: Backscattered electron SEM images of glass wafers and whole glass powders post-158 d *in situ* γ-irradiation dissolution (modified PCT-B) tests conducted at 40 °C under atmospheric conditions

The aqueous durability of the three glasses after 158 d in non-irradiated tests followed the trend, from most to least durable; POCO > ISG-2 > ISG-1 based on the NL_B_ (a conventional tracer of glass dissolution). This order changed for the 158 d *in situ* γ-irradiated tests from most to least durable: ISG-2 > POCO > ISG-1 (Fig. 2). Within experimental uncertainty, the glasses in both *in situ* γ-irradiated tests can be interpreted as exhibiting a similar rate of dissolution; however, the normalised mass loss of B, Na, Ca and Mg were slightly higher in γ-irradiated tests when compared to non-irradiated tests, whilst the normalised mass loss of Si was comparable or slightly lower. The NL_B_ of the ISG-1, ISG-2 and POCO glasses post-158 d *in situ* γ-irradiation dissolution was 0.87 ± 0.31, 0.60 ± 0.04 g m^–2^ and 0.68 ± 0.07 g m^–2^, respectively, compared to 0.62 ± 0.01, 0.57 ± 0.01 and 0.41 ± 0.06 in non-irradiated tests (Table 1; Fig. 2).

**Table 1:** Normalised mass loss of key elements from non-irradiated and irradiated studies after 158 d at 40 °C

Glass	Element	Non-irradiated samples 20 °C NL_*i*_ (g m^–2^)	Non-irradiated samples 40 °C NL_*i*_ (g m^–2^)	Irradiated samples 40 °C NL_*i*_ (g m^–2^)
ISG–1	B Na Si Ca	0.29 ± 0.00 0.22 ± 0.01 0.07 ± 0.00 0.07 ± 0.01	0.62 ± 0.01 0.61 ± 0.01 0.095 ± 0.004 0.098 ± 0.004	0.87 ± 0.31 0.71 ± 0.25 0.047 ± 0.004 0.15 ± 0.01
ISG–2	B Na Si Ca Mg	0.15 ± 0.00 0.15 ± 0.00 0.04 ± 0.00 0.13 ± 0.01 0.04 ± 0.01	0.57 ± 0.01 0.55 ± 0.01 0.088 ± 0.000 0.081 ± 0.010 0.11 ± 0.02	0.60 ± 0.04 0.57 ± 0.05 0.06 ± 0.01 0.31 ± 0.03 0.22 ± 0.01
POCO	B Na Si Ca Mg	0.16 ± 0.01 0.18 ± 0.02 0.02 ± 0.00 0.00 ± 0.00 0.13 ± 0.02	0.41 ± 0.06 0.50 ± 0.06 0.063 ± 0.000 0.067 ± 0.002 0.21 ± 0.00	0.68 ± 0.07 0.78 ± 0.07 0.07 ± 0.00 0.27 ± 0.05 0.54 ± 0.05

The final pH of the γ-irradiated leachate was approximately 1 pH unit lower than the baseline test leachates (Fig. 2), which is likely due to the production of H_3_O^+^ and H_2_O_2_ from the radiolysis of water. A pH decrease can account for the lower concentration of Si in γ-irradiated sample leachate as SiO_2_ solubility is higher under more alkaline conditions. A decrease in pH will promote ion exchange reactions between H_3_O^+^ and network-modifying cations in the glass alongside a significant increase in the solubility product of many cations increasing, Li et al. demonstrated that in acidified Na-silicate solutions, the conditional solubility product increased by approximately a factor of 100 with a single point decrease in pH [23]. The release of NL_Si_ and NL_Ca_ was near identical for ISG-1 in non-irradiated tests; however, in *in situ* γ-irradiated tests for ISG-1, the NL_Ca_ (0.16 ± 0.01 g m^–2^) was nearly a factor of three greater than the NL_Si_ (0.053 ± 0.004 g m^–2^). Similarly, NL_Si_ and NL_Ca_ were near identical for ISG-2 in non-irradiated tests (and NL_Mg_ was lower); however, NL_Ca_ and NL_Mg_ were higher than NL_Si_ in γ-irradiated tests. This observation supports the theory that the radiation-induced change in pH is affecting the dissolution of leachable, network-modifying elements and may also affect cation retention at the glass surface (surfaces are more negatively charged at higher pH and sorb positively charged cations more readily). Lemmens and Van Iseghem [14] also noticed an increase in leachable elements when borosilicate glass was exposed to γ-irradiation and attributed this to radiolytic acidification.

This study cannot rule out direct interaction of H_2_O_2_ produced by radiolysis with the glass surface. The literature reports higher H_2_O_2_ concentrations in the leachate of dissolved glass than in blank tests, which suggests an irradiation effect on the glass and its alteration layer [4]. However, even after two years of *in situ* γ-irradiation dissolution tests on SON68 glass (the French non-active simulant HLW glass) that received a 200 MGy total dose, defects and changes to glass and alteration layer were too few to observe a quantifiable effect on the residual dissolution rate (Stage II) [4].

Post-dissolution (γ-irradiated and baseline) SEM analysis did not reveal evidence for alteration layer formation at the resolution employed (Fig. 2). Future work focussing on similar dissolution tests but conducted at higher controlled temperatures and for longer duration should provide structural data on alteration layers formed under γ-irradiation. This will be helpful to further understand the mechanism of alteration layer and secondary phase development under extreme γ-irradiation environments [4]. A greater understanding of gel formation mechanisms under γ-irradiation, and the origin of H_2_O_2_ production will better aid future modelling. Determination of the concentration of H_2_O_2_ after irradiation would also aid in understanding of formation and consumption during the irradiation process particularly in determining radiolytic yield. Confidence in the negligible impact on the dissolution behaviour after a self-γ-irradiation dose of ~ 2,000 MGy expected for HLW glass after 10,000 years of disposal [12] may need to be assured for a robust safety case.

### Comparison with literature for ISG-1 and ISG-2

Previous studies on ISG-1 and ISG-2 have reported mixed results both regarding the effect of γ-irradiation and of the relative durability of the two glasses [24, 25]. As regards their performance under irradiation, Jiménez et al*.* [24] reported that the dissolution behaviour remained unchanged for pre-γ-irradiated and pristine glass PCT-B tests conducted on both the ISG-1 and ISG-2 at 90 °C for 7 d in UHQ water [24]. This test was notably much shorter than the experiment performed in this study. Interestingly, the relative behaviour of the two glasses at 40 °C and 20 °C (this study) was different to that observed at 90 °C by Jimenez et al. [24]. At 90 °C, it was reported that ISG-2 was less durable than ISG-1; however, the opposite appears to be true of long-term, low-temperature tests at least with respect to NL_B_. Near identical tests to those described in Jiménez et al. [24] were performed by Ryan et al*.* [16] at 90 °C but were conducted for 196 d where a divergence in the dissolution behaviour between ISG-1 and ISG-2 was observed. Reported NL_B_ for the ISG-1 and ISG-2 were ~ 2 and ~ 5 g m^–2^ after 196 d (pH_RT_ was measured at 9.0 ± 0.2 at all time points). These studies attribute the difference in the behaviour of ISG-1 and ISG-2 to the higher susceptibility of ISG-2 to dissolution because of the effect of adding MgO at the expense of CaO [16], where the mixture of the alkaline earths led to a deleterious effect [13, 24]. In this study, the NL_Ca_ and NL_Mg_ data for the ISG-2 show preferential leaching of Ca in irradiated samples (where the pH was lower) and at lower temperatures of 20 °C but not in non-irradiated samples at 40 °C. Surface layers were not visible (Fig. 2) and so it cannot be concluded if this meant retention of Mg in the glass or preferential incorporation of Mg in the alteration layer. The trend is reversed for the POCO glass, where NL_Mg_ is higher than NL_Ca_ in all systems which may be due to the fact that Ca is incorporated into an insoluble powellite (CaMoO_4_) crystalline phase [26, 27]. (Table 2).

**Table 2:** Comparison of available literature on γ-irradiation dissolution studies on ISG-1 and ISG-2

Study	Temperature	pH	Time (days)	Irradiation (MGy)	ISG-1 NL_B_ (g m^–2^) unless otherwise stated	ISG-2 NL_B_ (g m^–2^) unless otherwise stated
Jimenez et al*.* 2023 [24]	90 °C	9.0	7	None 0.95	2.15 ± 0.09 ppm 2.18 ± 0.02 ppm	4.31 ± 0.29 ppm 4.24 ± 0.38 ppm
Jimenez et al*.* 2022 [25]	90 °C	9.0	7	None 0.83 ~ 2	2.37 ± 0.04 2.35 ± 0.06 2.54 ± 0.05	– – –
Ryan et al. 2023 [16]	90 °C	9.0	198	None	0.89 ± 0.09	1.77 ± 0.2
This study	40 °C 40 °C 20 °C	9.0 8.0 9.1	158 158 158	None 21.6 None	0.62 ± 0.01 0.87 ± 0.31 0.29 ± 0.00	0.57 ± 0.01 0.60 ± 0.04 0.15 ± 0.00

### Comparison with literature for POCO glass

The NL_B_ obtained from the POCO HLW glass after 158 d (0.40 ± 0.06 g m^–2^ non-irradiated and 0.68 ± 0.07 g m^–2^ irradiated) are similar to the 0.75 ± 0.08 g m^–2^ value report by Fisher & Corkhill [18] from their 168 d final sampling time point of a PCT-B test conducted under anoxic conditions in Ca(OH)_2_ solution at 40 °C with a S/V of 1,200 m^–1^, where the PH_RT_ post-test measured 12.7. However, the authors note that clumping of the glass powders in the hyperalkaline tests likely led to an underestimation of the chemical durability. Alteration layers were also not observed in the hyperalkaline tests where dissolution progressed at the residual rate (Stage II) at the time of sampling. POCO glass contains a variety of crystalline phases: powellite (CaMoO_7_), ruthenium dioxide (RuO_2_), zincochromite (ZnCr_2_O_4_), zircon (ZrSiO_4_) and cerianite (Ce_0.6_Zr_0.4_O_2_) [19], and it has been determined that powellite and zircon become amorphous and swell considerably after Ni and Au ion irradiation simulating α-recoil damage in active POCO HLW. It is possible that POCO HLW may develop microcracks resulting from radiation exposure (and cannister cooling), thus, increasing the available surface area for radionuclide release [28]. Subjected to a dissolving media, cracks and microcracks are known to become supersaturated and readily form alteration products [29, 30]. Future corrosion studies on fractured/coarse surface glass subjected to *in situ* radiation (α or γ) dissolution tests may be useful to fundamentally explore the alteration layer development to better understand and predict the long-term durability of disposed HLW-type glasses, not just POCO, in a geological repository.

## Conclusion

*In situ* γ-irradiated PCT-B-type dissolution tests were conducted on the ISG (1 & 2) and UK POCO HLW glasses. Results showed a slight increase in the normalised mass loss of all elements when compared to non-irradiated control studies conducted on the same glasses. This difference was tentatively attributed to acidification associated with radiolysis during γ-irradiation and is evidenced by a measurable decrease in the pH and a change in the relative leach rates of elements from the glass associated with dissolution in a more acidic regime. Interestingly, the relative durabilities of ISG-1 and ISG-2 were reversed in these lower temperature dissolution tests compared to those performed at 90 °C with ISG-2 showing slightly higher durability than ISG-1. Post-dissolution, samples were characterised by SEM and did not show evidence for alteration layer formation at the resolution employed; thus, the effect of *in situ* γ-irradiation on alteration layer development was not determined. Dissolution data from both tests provide a reference for future studies aimed at furthering the understanding of the effect of γ-irradiation on glass dissolution and may provide model input parameters to aid the development of glass corrosion models pertinent to the field of radioactive waste disposal.

## Data Availability

All presented data (included raw ICP-MS elemental concentration data) are available upon request.
